# Improved precision of noise estimation in CT with a volume-based approach

**DOI:** 10.1186/s41747-021-00237-x

**Published:** 2021-09-10

**Authors:** Hendrik Joost Wisselink, Gert Jan Pelgrim, Mieneke Rook, Ivan Dudurych, Maarten van den Berge, Geertruida H. de Bock, Rozemarijn Vliegenthart

**Affiliations:** 1 Department of Radiology, EB44, University Medical Center Groningen, University of Groningen, Hanzeplein 1, 9713 GZ Groningen, The Netherlands; 2 Department of Radiology and Nuclear Medicine, Martini Hospital Groningen, Groningen, The Netherlands; 3 Department of Pulmonology, Groningen Research Institute for Asthma and COPD (GRIAC), University Medical Center Groningen, University of Groningen, Groningen, The Netherlands; 4 Department of Epidemiology, University Medical Center Groningen, University of Groningen, Groningen, The Netherlands

**Keywords:** Data accuracy, Noise, Pulmonary disease (chronic obstructive), Thorax, Tomography (x-ray computed)

## Abstract

Assessment of image noise is a relevant issue in computed tomography (CT). Noise is routinely measured by the standard deviation of density values (Hounsfield units, HU) within a circular region of interest (ROI). We explored the effect of a spherical volume of interest (VOI) on noise measurements. Forty-nine chronic obstructive pulmonary disease patients underwent CT with clinical protocol (regular dose [RD], volumetric CT dose index [CTDIvol] 3.04 mGy, 64-slice unit), and ultra-low dose (ULD) protocol (median CTDIvol 0.38 mGy, dual-source unit). Noise was measured in 27 1-cm^2^ ROIs and 27 0.75-cm^3^ VOIs inside the trachea. Median true noise was 21 HU (range 17-29) for RD-CT and 33 HU (26-39) for ULD-CT. The VOI approach resulted in a lower mean distance between limits of agreement compared to ROI: 5.9 *versus* 10.0 HU for RD-CT (−40%); 4.7 *versus* 9.9 HU for ULD-CT (−53%). Mean systematic bias barely changed: −1.6 *versus* −0.9HU for RD-CT; 0.0 to 0.4HU for ULD-CT. The average measurement time was 6.8 s (ROI) *versus* 9.7 (VOI), independent of dose level. For chest CT, measuring noise with a VOI-based instead of a ROI-based approach reduces variability by 40-53%, without a relevant effect on systematic bias and measurement time.

## Key points

- Volume-based noise measurement increased precision compared to a circular region of interest.
- For regular and ultra-low dose chest CT, mean variability decreased by 40−53%.
- Volume-based measurements did not take substantially longer time than the conventional method.

## Background

In computed tomography (CT) imaging, the call for dose reduction has led to ongoing efforts to mitigate the effects of increased noise. Current strategies include iterative reconstruction methods and artificial intelligence-based techniques. Less attention is given to optimization of noise measurement. The common definition of image noise is the standard deviation (SD) of the measured Hounsfield units (HU) in a physically homogeneous volume [1]. The noise level depends on the specific acquisition and reconstruction parameters, total attenuation of the scan subject, absolute density of the tissue of interest, and on the location in the scanner bore (*i.e.*, the distance of a given voxel to the center of the field of view). For that reason, it is important to measure a calibration structure with a density and location similar to the tissue of interest. By using a standardized location, the noise measurement provides a good indication for inherent image noise, except in cases of local image artifacts like beam hardening [2, 3].

In chest CT, optimal representation of image noise may be obtained by segmenting the entire tracheobronchial tree lumen, and measuring the SD of this air. However, this is not feasible in most clinical software programs, due to software limitations and/or time constraints. Because of this, the current clinical practice is to measure the SD in a 1-cm^2^ circular region of interest (ROI) inside the trachea [2, 4]. Accurate noise measurements are important for protocol optimization and quantification processes [5–7]. For instance, in emphysema quantification by CT lung densitometry, image noise may affect the threshold needed for reliable distinction between emphysema and normal lung tissue [2, 5].

Moreover, reducing variability of HU measurements may have other clinical implications. The ROI-based technique is commonly used for the assessment of liver parenchyma density and for kidney stone density. These measurements, too, are prone to variation, partly inherent to the ROI-based approach and exacerbated by the sensitivity of mean to outliers [6, 7]. This suggests that the results of this study are applicable to more CT scan indication than just lung CT imaging and assessment of noise. Since reproducibility largely depends on the number of voxels included in the calculation, using a volume-based approach with a volume of interest (VOI) may result in greater precision, without requiring more complicated processing (*e.g.*, by measuring multiple ROIs). Despite this, many studies over the years, including recent studies, have used an ROI-based approach [8–12].

The aim of this study was to determine the systematic bias and variability of ROI-based and VOI-based noise measurements in CT scans obtained at two radiation doses, regular dose (RD) and ultra-low dose (ULD), resulting in low and high noise levels, respectively. These two study arms were independently analyzed.

## Methods

### Patient cohort

In an on-going chronic obstructive pulmonary disease (COPD) patient study, 50 patients underwent non-contrast clinical chest CT at RD as well as ULD CT between February 2018 and June 2018. The two scans were made on the same day and the order was randomized between participants. The institutional ethical board gave approval for this study and participants provided written informed consent (METC 2015/335, clinicaltrials.gov NCT02477397). Table 1 shows a summary of the patient characteristics. One patient was excluded due to a body habitus far outside the normal range for COPD patients: a body mass index of 56, over 5 standard deviations (SDs) above the mean of the remainder of the cohort.

**Table 1:** Patient cohort characteristics (*n* = 49)

Characteristic	Value
Age (years)	66 ± 7
Sex	34 males (69%), 15 females (31%)
Body mass index (kg/m^2^)	28.0 ± 5.3
FEV_1_ (% of predicted)	53 ± 16
FEV_1_/FVC (%)	42.4 ± 11.2
CTDIvol (mGy), regular CT protocol	3.04
DLP (mGy × cm), regular CT protocol	105.1 (86.3−134.1)
CTDIvol (mGy), ultra-low dose CT protocol	0.38 (0.19−1.06)
DLP (mGy × cm), ultra-low dose CT protocol	16.6 (7.3−29.8)

Values are given as mean ± standard deviation or median (range), unless stated otherwise. To facilitate comparison, the DLP for the regular-dose CT is expressed as median (range), despite a normal distribution (*p* = 0.103). *CT* computed tomography, *FEV*_*1*_ forced expiratory volume in 1 s; *FVC* forced vital capacity, *CTDIvol* volumetric CT dose index, *DLP* dose length product

### CT scans

The RD-CT scans were acquired on a routine 64-slice CT system (Somatom Definition AS, Siemens Healthineers, Forchheim, Germany) with routine high-resolution CT protocol of 40 mAs (fixed tube current) and 120 kVp (volumetric CT dose index [CTDIvol] 3.04 mGy). The ULD-CT scans were acquired on a third generation dual-source CT system (Somatom Force, Siemens Healthineers, Forchheim, Germany) with 70 mAs (reference tube current), at 100 kVp with Sn filter (median CTDIvol 0.38 mGy, range 0.19-1.34 mGy). The pitch was 1.5 for RD-CT and 1.6 for ULD-CT. The field of view was adjusted to the individual patient size for each scan (range 317−500 mm). Scans were reconstructed with slice thickness/increment of 1.0/0.7 mm, filtered back projection and a soft kernel. The two kernels used B30f and Br40, respectively, are suggested by the vendor as similar and are generally treated in literature as comparable [11].

### Image analysis

**Fig. 1:**
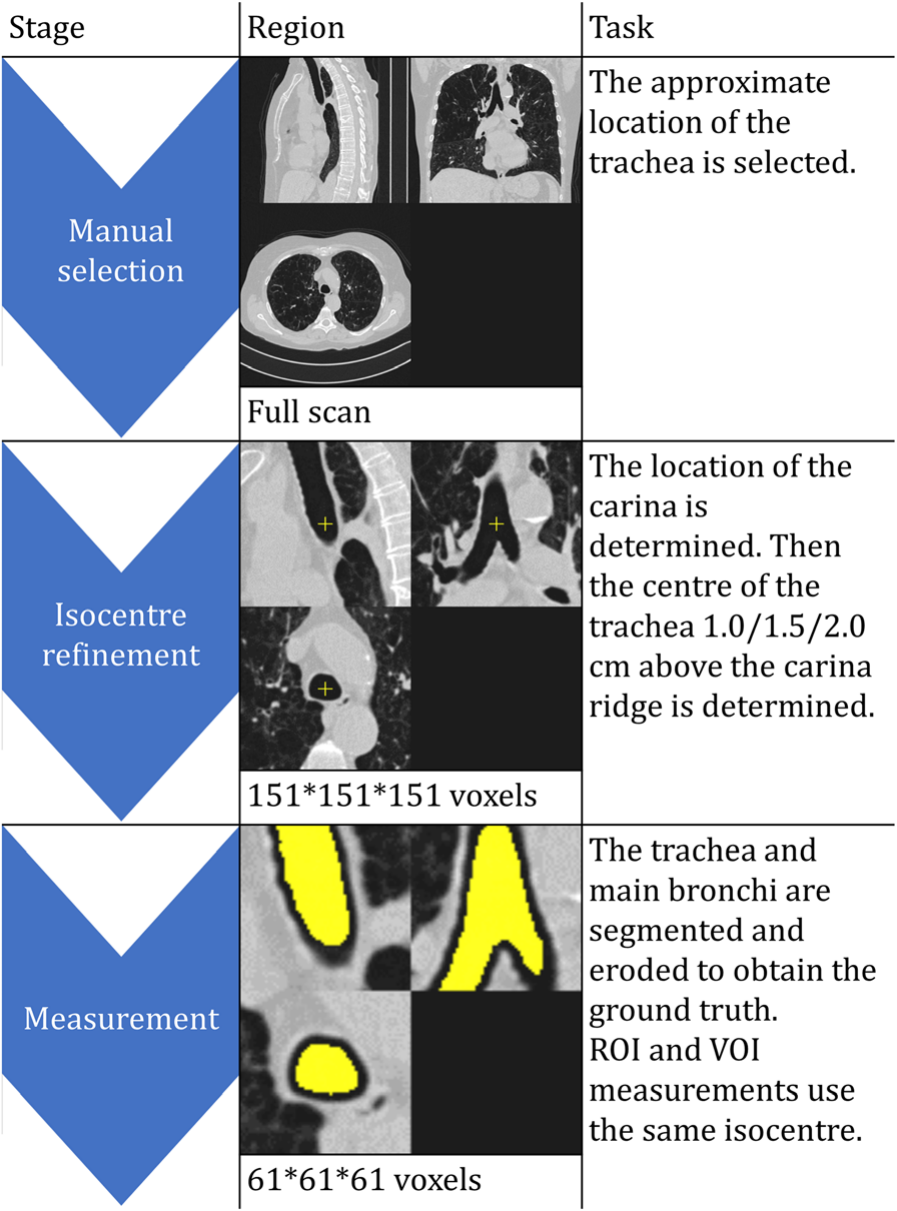
Flow chart of the steps to determine the ground truth noise and the isocenter for the measurements. ROI, region of interest; VOI, volume of interest

Analysis was performed with an in-house developed MATLAB script (MATLAB R2020b, The Mathworks, Natick, MA, USA). The complete function is available online via http://tiny.cc/YL3BNUQ4. The choice for a stand-alone analysis script was made to avoid time-consuming efforts to determine the variability of manual measurements. The simulation method is a best-case scenario for what a human reader would achieve. The noise level was defined as the SD of the selected voxels. To obtain the ground truth for the noise level for intra-thoracic air, a section of the tracheobronchial tree (caudal trachea and proximal bronchi) was segmented in a 61 × 61 ×61 voxel region (referred to as *trachea segmentation* or *segmentation* in the remainder of this paper). Due to the patient-specific field of view, the size in millimeter of this cubic region differed case by case. See the flow chart in Fig. 1 for a description of each step in this process. For the ROI and VOI, a standardized measurement location was used (a fixed distance above the carina ridge). The edge of the segmentation was removed with a morphological erosion (a mathematical operation removing boundary pixels) to avoid edge artifacts.

To simulate repeated manual measurements, a jitter was applied, meaning the centroid was moved one voxel in *x*, *y*, and *z*-direction, resulting in 27 possible locations. For all 27 centroids, the noise was measured with both a circular ROI and spherical VOI. The radius was based on an area of 1.0 cm^2^, resulting in a VOI of approximately 0.75 cm^3^. Due to these definitions, the number of voxels used for these analyses depended on the FOV and the slice thickness. For the ROI, between 101 and 261 voxels were included (median 177 voxels), for the VOI between 1117 and 2789 voxels (median 1849 voxels). If either the ROI or VOI contained voxels outside, the segmentation (prior to the previously mentioned morphological erosion), both ROI and VOI were excluded from further analysis for that measurement position, mimicking manual measurements. The values obtained at the level above the carina ridge that resulted in the fewest rejections was used for the remainder of the analysis (at either 1.0, 1.5, or 2.0 cm), to further mimic a manual measurement accounting for anatomical variation. This height selection was done separately for each scan.

To estimate the extra time required for a VOI-based measurement, a trained researcher (HJW) measured the noise ten times manually with each strategy. The Syngo.Via software (version VB40A, Siemens Healthineers, Forcheim, Germany) was used to perform the measurements. To account for the imprecision of a manual measurement and considering that a precise area or volume may not be possible given the voxel size of a specific scan, a radius difference of up to 5% with the area or volume described below was considered acceptable when measuring the noise. The order of the measurements was randomized.

### Statistical analysis

Statistical analysis was performed with MATLAB R2020b (The Mathworks, Natick, MA, USA). Bland-Altman analysis was used to determine the systematic bias between the true noise level and measured noise [13]. The difference between the systematic biases of the two measurement strategies was tested with the Wilcoxon signed-rank sum test. Variability was defined as the distance between the limits of agreement. Because this is directly related to the variance, Levene’s test was used. Each characteristic in Table 1 (except sex) was tested separately for normality with the Shapiro-Wilk test.

## Results

**Fig. 2:**
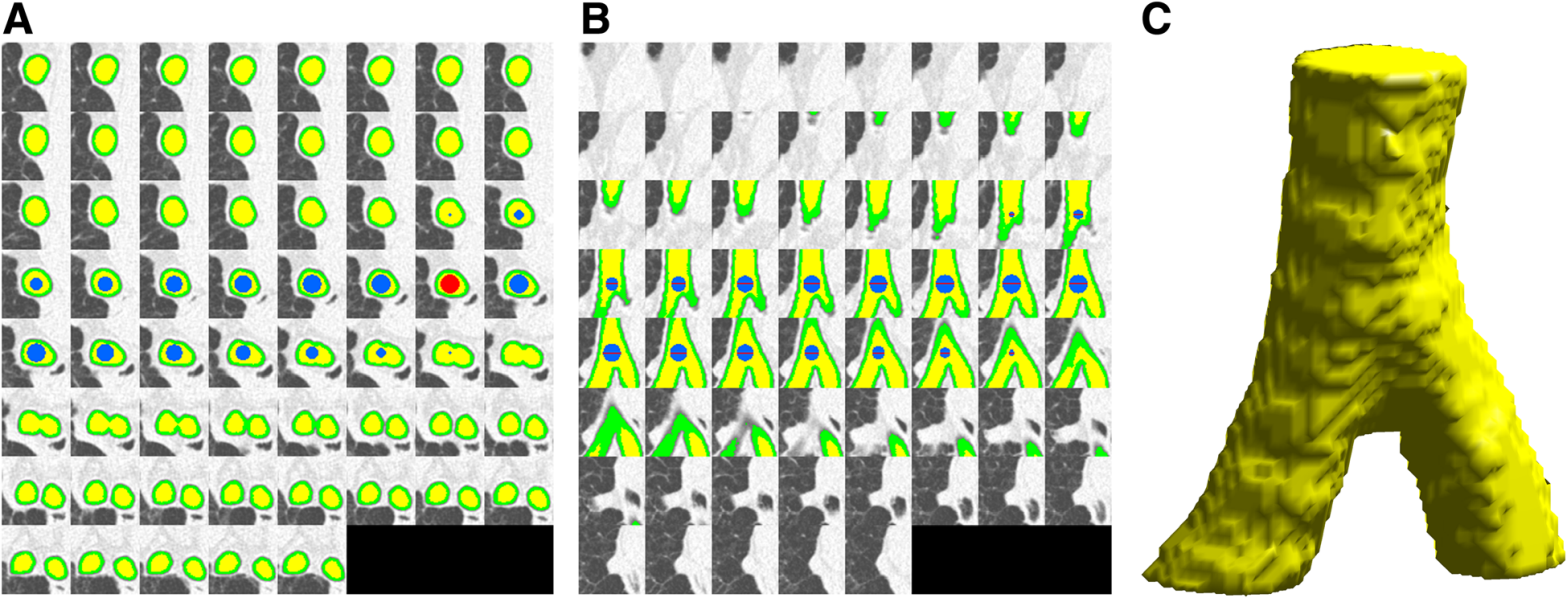
Subsection of the CT images around the carina (window width 1600 HU, window level −700 HU). The red part is the position of the region of interest, the blue is the volume of interest, the yellow is used to measure the ground truth, and the green area was removed from the segmentation to prevent edge artifacts like the partial volume effect. This image shows the measurement with the isocenter 1.0 cm above the carina ridge. **a** Axial images. **b** Coronal images, interpolated to account for the anisotropic dimensions of the voxels. **c** Volume render of the yellow segmentation

The seed point location and the segmentation of the air in the trachea was visually confirmed for each reconstruction. One representative case is depicted in Fig. 2, showing successful segmentation without excluding large parts of the trachea or main bronchi, or including parenchyma or bronchial wall. For RD-CT, 66 of 1323 jitter-scan combinations (5.0%) were discarded because the ROI or VOI contained voxels outside the trachea. For ULD-CT, 84 of 1323 combinations (6.3%) were discarded. This led to a total exclusion rate of 150 of 2646 values (5.7%). The range of true noise based on the trachea segmentation was 17−29 HU for RD-CT and 26−39 HU for ULD-CT. As these ranges are based on the true noise, only a single value per patient has been obtained. For the ranges of the noise measured with a ROI or a VOI, all valid measurements were considered. The range of noise measured with a ROI was 11−32 HU for RD-CT (based on 1257 measurements) and 23−44 HU for ULD-CT (based on 1239 measurements). The respective ranges for the VOI-based measurement were 13−30 HU for RD-CT and 25−43 HU for ULD-CT.

**Fig. 3:**
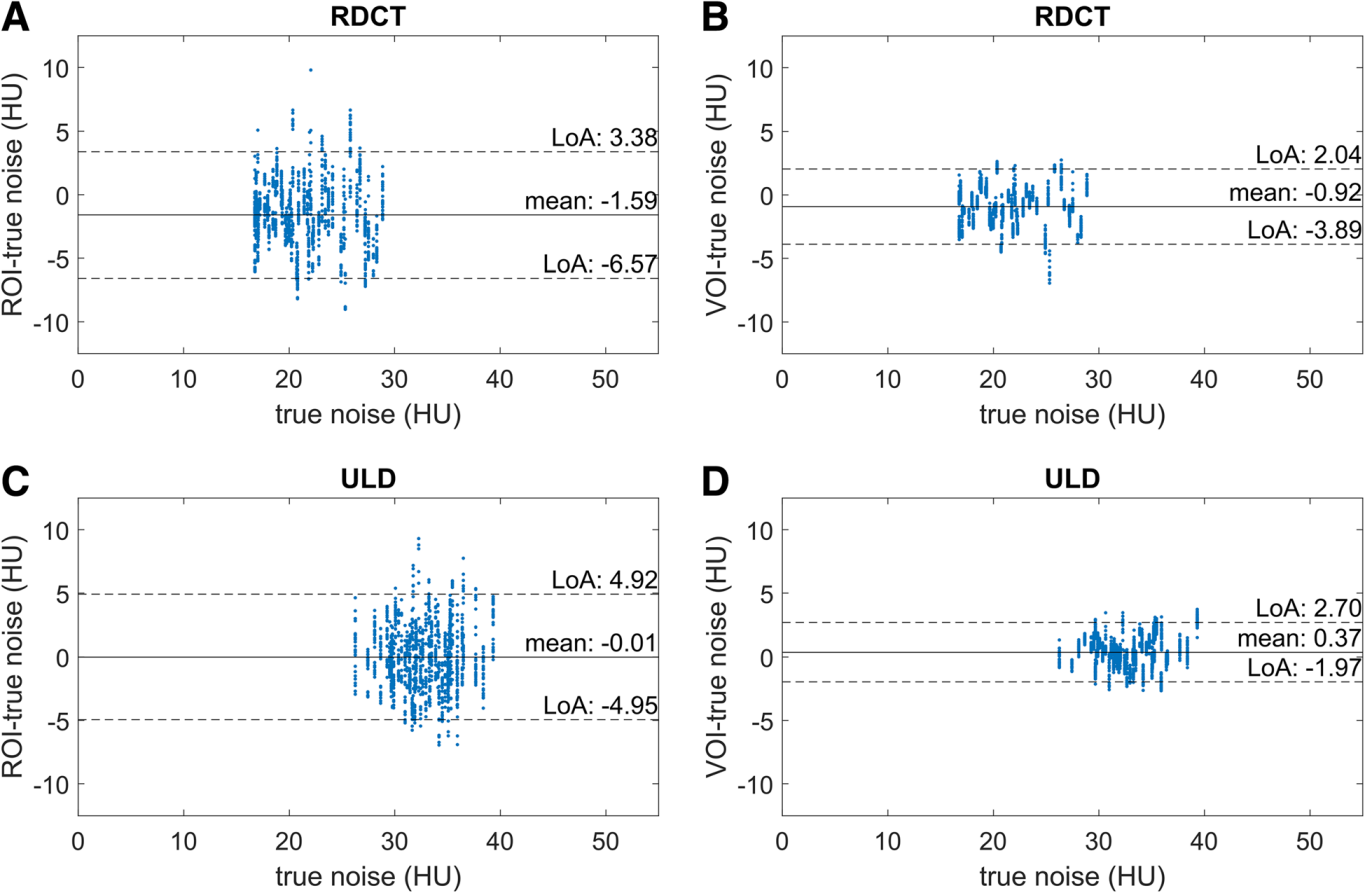
Results of the Bland-Altman analyses. Each plot shows the difference between the noise measured with either ROI or VOI and ground truth noise on the *y*-axis, versus ground truth on the *x*-axis. Regular radiation dose computed tomography protocol measured with an ROI (**a**) or a VOI (**b**), same data for ultra-low dose protocol (**c** and **d**, respectively). ROI, region of interest; VOI, volume of interest; LoA, limits of agreement; HU, Hounsfield units

The results of the Bland-Altman analysis in residual plots are shown in Fig. 3. As the noise was measured in 27 different locations, there are multiple dots for each scan. Because every scan has only one ground truth noise value, this results in vertical patterns. For the VOI-based approach, the distance between limits of agreement, compared to the ROI-based approach, decreased from 10.0 to 5.9 for RD-CT (40% reduction, *p* < 0.001) and from 9.9 to 4.7 for ULD-CT (53% reduction, *p* < 0.001), indicating a lower inter-measurement variation when using the VOI-based method. There was a minimal effect on the systematic bias for both the RD-CT (−1.6 to −0.9 HU, *p* < 0.001) and ULD-CT (0.0 to 0.4 HU, *p* < 0.001).

The manual ROI measurement by the trained researcher took 6.8 s on average; for the VOI measurement, this increased by 2.9 s to 9.7 s (+43%) and would therefore not meaningfully increase the time required to read a CT scan.

## Discussion

In this study, we showed that a VOI-based noise measurement approach significantly improves precision compared to a ROI-based approach, especially in CT scans with a higher intrinsic noise level, without a relevant trade-off in terms of measurement time.

As early as 1978, an alternative method for objective measurement of image noise was published: a noise power spectrum (NPS) [14]. This has the benefit of not relying on the measured region being homogeneous and of providing a more detailed description of noise, instead of relying on a single descriptive value. Despite the NPS method being available for decades, clinical studies have continued to use the ROI method [8–12] while the NPS method is only used in highly technical applications [15]. To our knowledge, no clinical system provides the option to compute the NPS. Thus, the calculation of the NPS will most likely require exporting the scans for external processing, making it less desirable for either research or clinical use. This same limitation applies to using the segmented trachea to measure the noise.

Other studies proposed other methods to improve on the ROI-based method, *e.g.*, by subtracting two adjacent slices (similar to how digital subtraction angiography works) before calculating either a local (pixel-by-pixel) SD, a regional SD, or multiple regional SDs [16–19]. Such methods are particularly useful in situations where noise does not have a Gaussian distribution, or where pixel value differences exist due to anatomical structures [17, 18]. Another commonly proposed method is to average multiple regions [16, 17]. This is mostly used for liver parenchyma, where multiple smaller ROIs are sometimes used to ensure a measurement area that better reflects the organ as a whole [8]. To our knowledge, none of the previously mentioned alternatives to the ROI-based method are available for routine clinical use.

Given the increased use of artificial intelligence (AI), any specific application of a ROI-based measurement may eventually be replaced by an AI tool. Such tools may forgo measuring a specific density or noise level in favor of directly assessing the intended biomarker [20, 21]. Up to the moment that an AI tool (for this specific application) does become available, the VOI-based method proposed in this work is a simple and quick option, to be preferred over ROI evaluation.

The potentially quick and easy applicability is one of the main advantages of using a volume-based approach, which may help implementation in both research and clinical practice. A VOI-based measurement should be widely available in PACS reading systems, often in the same drop-down menu as the ROI-based measurement option. The extra time required is limited.

More generally, volumetric analyses on CT scans are increasingly common. An example of this is the volumetric assessment of lung nodules, which increasingly replaces the diameter-based approach [22]. Additionally, some nuclear medicine guidelines also require the use of volumetric measurements [23]. To our knowledge, only one previous study has focused on the use of volume-based noise measurements in radiology [24], outside of recent technical quality standards like the QIBA lung density profile [25]. This is unfortunate, as the applicability is likely not limited to measuring noise, but may also extend to other situations where a density measurement is performed, *e.g.*, when measuring liver density or muscle density [8, 11]. Future research should be conducted to confirm this expectation.

Some aspects of this study may potentially limit the generalizability of these results. The scans were made on CT systems from one vendor only in a relatively small COPD patient cohort, without including healthy controls. However, only testing scans from a single vendor is not expected to influence the conclusion. To improve generalizability of the results, scans were acquired with many differences in the scan protocol like radiation spectrum, mAs, and reconstruction kernel. Importantly, the aim of our study was not to compare noise between an RD and an ULD CT scan protocol, but to investigate the method to quantify the noise. This means the scans should not be analyzed as pairs, but should be treated as two study arms that are independently analyzed. The results from both scanners support the same conclusion, even with the different scan protocols. The small size of the cohort is unlikely to affect the conclusion, even if a larger cohort size would further increase confidence in quantifying the difference between the two methods. Similarly, there is no technical reason why the presence or absence of COPD would influence the noise characteristics in the trachea of an ROI compared to a VOI. Lastly, switching from an automated script to a human reader is unlikely to substantially change the results.

In conclusion, in chest CT protocols, measuring image noise with a VOI-based approach instead of a ROI-based approach reduces variability by 40-53%, without a relevant effect on systematic bias and measurement time.

## Data Availability

The complete code for the main analysis is available online via http://tiny.cc/YL3BNUQ4.

## Abbreviations

COPD: Chronic obstructive pulmonary disease
CT: Computed tomography
CTDIvol: Volumetric CT dose index
HU: Hounsfield units
NPS: Noise power spectrum
RD: Regular dose
SD: Standard deviation
ULD: Ultra-low dose
VOI: Volume of interest

## References

[1] European Commission (2000) European guidelines on quality criteria for CT, available at https://op.europa.eu/s/n8PM, archived at http://web.archive.org/web/20210225144451/https://op.europa.eu/o/opportal-service/download-handler?identifier=d229c9e1-a967-49de-b169-59ee68605f1a&format=pdf&language=en&productionSystem=cellar. Office for Official Publications of the European Communities

[2] Messerli M, Ottilinger T, Warschkow R, Leschka S, Alkadhi H, Wildermuth S, Bauer RW (2017). Emphysema quantification and lung volumetry in chest X-ray equivalent ultralow dose CT–intra-individual comparison with standard dose CT. Eur J Radiol.

[3] den Harder AM, de Boer E, Lagerweij SJ, Boomsma MF, Schilham AMR, Willemink MJ, Milles J, Leiner T, Budde RPJ, de Jong PA (2018). Emphysema quantification using chest CT: influence of radiation dose reduction and reconstruction technique. Eur Radiol Exp.

[4] Martin SP, Gariani J, Feutry G, Adler D, Karenovics W, Becker CD, Montet X (2019). Emphysema quantification using hybrid versus model-based generations of iterative reconstruction: SAFIRE versus ADMIRE. Medicine.

[5] Wisselink HJ, Pelgrim GJ, Rook M, van den Berge M, Slump K, Nagaraj Y, van Ooijen P, Oudkerk M, Vliegenthart R (2019). Potential for dose reduction in CT emphysema densitometry with post-scan noise reduction: a phantom study. Br J Radiol.

[6] Ganeshan B, Miles KA, Young RC, Chatwin CR (2007). In search of biologic correlates for liver texture on portal-phase CT. Acad Radiol.

[7] Bora A, Alptekin C, Yavuz A, Batur A, Akdemir Z, Berköz M (2014). Assessment of liver volume with computed tomography and comparison of findings with ultrasonography. Abdominal Imaging.

[8] Kalra MK, Maher MM, Kamath RS, Horiuchi T, Toth TL, Halpern EF, Saini S (2004). Sixteen–detector row CT of abdomen and pelvis: study for optimization of z-axis modulation technique performed in 153 patients. Radiology.

[9] Pontana F, Pagniez J, Flohr T, Faivre JB, Duhamel A, Remy J, Remy-Jardin M (2011). Chest computed tomography using iterative reconstruction vs filtered back projection (Part 1): evaluation of image noise reduction in 32 patients. Eur Radiol.

[10] Wetzl M, May MS, Weinmann D, Hammon M, Treutlein C, Zeilinger M, Kiefer A, Trollmann R, Woelfle J, Uder M, Rompel O (2020). Dual-source computed tomography of the lung with spectral shaping and advanced iterative reconstruction: potential for maximum radiation dose reduction. Pediatr Radiol.

[11] Lenga L, Lange M, Martin SS, Albrecht MH, Booz C, Yel I, Arendt CT, Vogl TJ, Leithner D (2021). Head and neck single-and dual-energy CT: differences in radiation dose and image quality of 2nd and 3rd generation dual-source CT. Br J Radiol.

[12] Noda Y, Iritani Y, Kawai N, Miyoshi T, Ishihara T, Hyodo F, Matsuo M (2021) Deep learning image reconstruction for pancreatic low-dose computed tomography: comparison with hybrid iterative reconstruction. Abdom Radiol:1–7. 10.1007/s00261-021-03111-x 10.1007/s00261-021-03111-x 33973060

[13] Bland JM, Altman DG (1986). Statistical methods for assessing agreement between two methods of clinical measurement. Lancet.

[14] Riederer SJ, Pelc NJ, Chesler DA (1978). The noise power spectrum in computed X-ray tomography. Phys Med Biol.

[15] Pan T, Hasegawa A, Luo D, Wu CC, Vikram R (2020). Technical note: impact on central frequency and noise magnitude ratios by advanced CT image reconstruction techniques. Med Phys.

[16] Christianson O, Winslow J, Frush DP, Samei E (2015). Automated technique to measure noise in clinical CT examinations. Am J Roentgenol.

[17] Tian X, Samei E (2016). Accurate assessment and prediction of noise in clinical CT images. Med Phys.

[18] Goerner FL, Clarke GD (2011). Measuring signal-to-noise ratio in partially parallel imaging MRI. Med Phys.

[19] Malkus A, Szczykutowicz TP (2017). A method to extract image noise level from patient images in CT. Med Phys.

[20] Yi X, Guan X, Chen C, Zhang Y, Zhang Z, Li M, Liu P, Yu A, Long X, Liu L, Chen BT, Zee C (2018). Adrenal incidentaloma: machine learning-based quantitative texture analysis of unenhanced CT can effectively differentiate sPHEO from lipid-poor adrenal adenoma. J Cancer.

[21] Masuda T, Nakaura T, Funama Y, Okimoto T, Sato T, Higaki T, Noda N, Imada N, Baba Y, Awai K (2019). Machine-learning integration of CT histogram analysis to evaluate the composition of atherosclerotic plaques: validation with IB-IVUS. J Cardiovasc Comput Tomogr.

[22] Han D, Heuvelmans MA, Oudkerk M (2017). Volume versus diameter assessment of small pulmonary nodules in CT lung cancer screening. Transl Lung Cancer Res.

[23] Boellaard R, Delgado-Bolton R, Oyen WJ, Giammarile F, Tatsch K, Eschner W, Verzijlbergen FJ, Barrington SF, Pike LC, Weber WA, Stroobants S, Delbeke D, Donohoe KJ, Holbrook S, Graham MM, Testanera G, Hoekstra OS, Zijlstra J, Visser E, Hoekstra CJ, Pruim J, Willemsen A, Arends B, Kotzerke J, Bockisch A, Beyer T, Chiti A, Krause BJ, European Association of Nuclear Medicine (EANM) (2015). FDG PET/CT: EANM procedure guidelines for tumour imaging: version 2.0. Eur J Nucl Med Mol Imaging.

[24] Jensen CT, Liu X, Tamm EP, Chandler AG, Sun J, Morani AC, Javadi S, Wagner-Bartak NA (2020). Image quality assessment of abdominal CT by use of new deep learning image reconstruction: initial experience. Am J Roentgenol.

[25] QIBA Lung Density Biomarker Committee. Computed tomography: lung densitometry, quantitative imaging biomarkers alliance. Profile Stage: consensus. September 04, 2020. Available from: http://web.archive.org/web/20201216134027/https://qibawiki.rsna.org/images/a/a8/QIBA_CT_Lung_Density_Profile_090420-clean.pdf

